# Supplementary material for: The interactome of intact mitochondria by cross-linking mass spectrometry provides evidence for coexisting respiratory supercomplexes
Source: Mol Cell Proteomics. 2017 Dec 8;17(2):216–32. doi: 10.1074/mcp.RA117.000470 (PMC5795388; doi:10.1074/mcp.RA117.000470)
Supplement: Supplemental Data [file supp_RA117.000470_133922_0_supp_23978_fzffwf.zip › spectra_annotation/mito_DR_spectra_annotation/465-1-12-1-4-1.pdf]

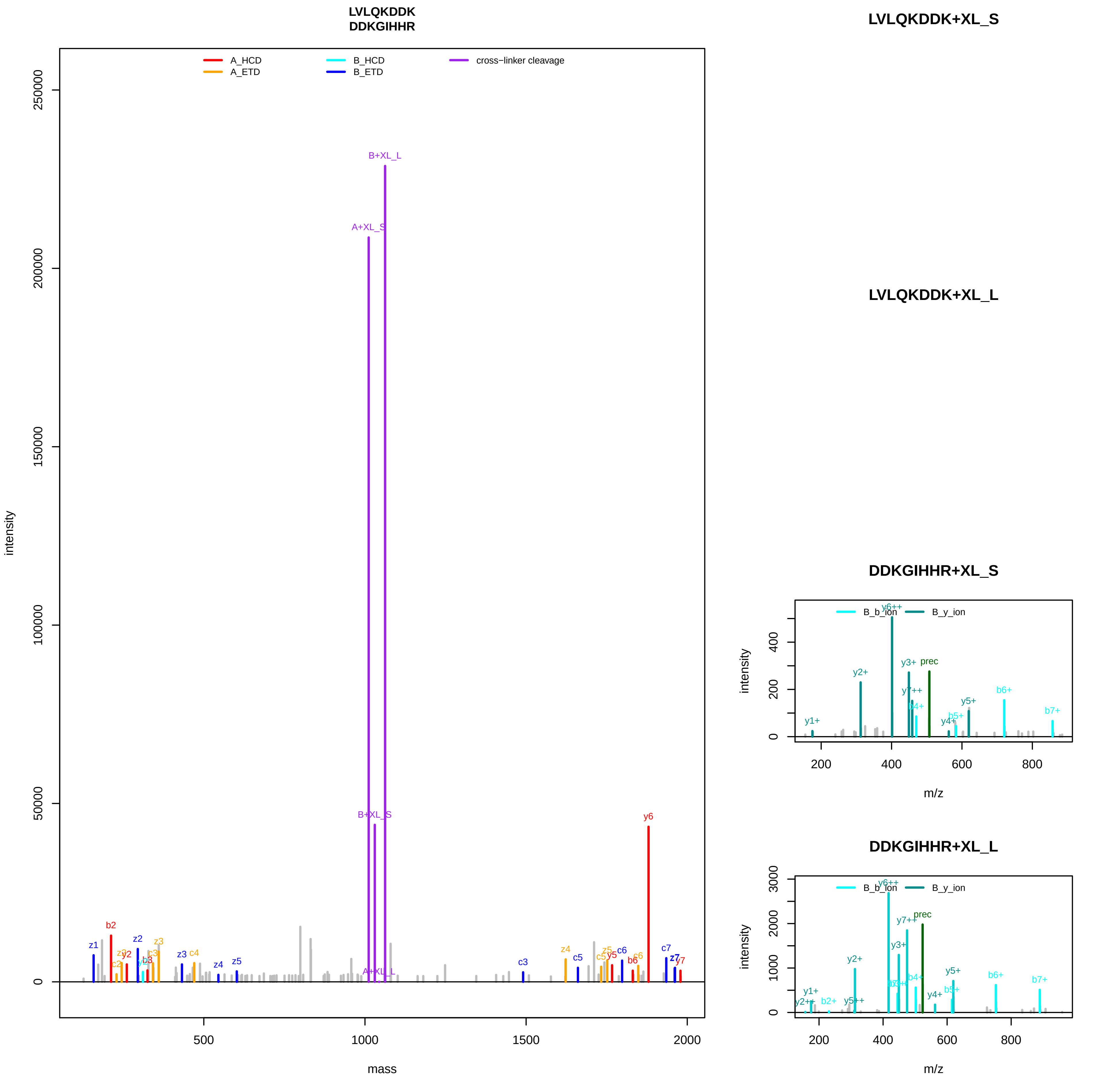
LVLQKDDK
DDKGIHHR
A_HCD
A_ETD
B_HCD
B_ETD
cross−linker cleavage
B+XL_L
A+XL_S
B+XL_S
A+XL_L
y6
b2
z1
z2
z3
c4
z4
z5
c3
c5
c6
c7
z7
y7
b6
y5
c2
y2
b3
intensity
mass
0
50000
100000
150000
200000
250000
500
1000
1500
2000
LVLQKDDK+XL_S
LVLQKDDK+XL_L
DDKGIHHR+XL_S
DDKGIHHR+XL_L
B_b_ion
B_y_ion
y6++
y3+
prec
y2+
y7++
b4+
b5+
y5+
b6+
b7+
y1+
y4+
m/z
200
400
600
800
1000
2000
3000
